# Recent advances in understanding antitumor immunity

**DOI:** 10.12688/f1000research.9356.1

**Published:** 2016-10-20

**Authors:** Rodrigo Ramella Munhoz, Michael Andrew Postow

**Affiliations:** 1 Melanoma/Sarcoma Group, Oncology Center, Hospital Sírio Libanês, São Paulo, Brazil; 2 Melanoma and Immunotherapeutics Service, Memorial Sloan Kettering Cancer Center, New York, NY, USA; 3 Weill Cornell Medical College, New York, NY, USA

**Keywords:** antitumour immunity, immune response, tumour immunotherapies

## Abstract

The term “antitumor immunity” refers to innate and adaptive immune responses which lead to tumor control. Turning the immune system into a destructive force against tumors has been achieved in a broad range of human cancers with the use of non-specific immunotherapies, vaccines, adoptive-cell therapy, and, more recently with significant success, through blockade of immune checkpoints. Nevertheless, the efficacy of these approaches is not universal, and tools to identify long-term responders and primarily refractory patients are warranted. In this article, we review recent advances in understanding the complex mechanisms of antitumor immunity and how these developments can be used to address open questions in a setting of growing clinical indications for the use of immunotherapy.

## Introduction

Using the immune system to fight cancer has been confirmed as one of the major breakthroughs in oncology, yielding the possibility of long-term clinical benefit and prolonged survival. Despite the recent advances with immune checkpoint-directed approaches, the concept of “immunotherapy” dates back to the 19 ^th^ and early 20 ^th^ century with Wilhelm Busch, William B. Coley, and Paul Ehrlich and comprises distinct strategies, including vaccines, non-specific cytokines, and adoptive cell therapies ^1^. The introduction of monoclonal antibodies targeting co-receptors of immune activation resulted in unprecedented benefits in the management of distinct malignancies, with exceptional results in melanoma, renal cell carcinoma, Merkel cell carcinoma, lung cancer, urothelial carcinoma, and other neoplasms ^2– 7^.

Nevertheless, despite the certainties already available that are redefining the landscape of cancer treatment, several questions emerged to daunt clinicians and scientists: how do we select the best candidates for therapy? What factors are involved in primary and acquired resistance? What are the best biomarkers to guide treatment decisions and rationalize costs? How do we pick the best combinations to optimize outcomes?

Elucidating the mechanisms regulating the interactions between the immune system and cancer cells is critical in order to provide tools to address the growing number of open questions, overcome resistance, and broaden the benefits of immunotherapy to more patients.

## The tumor-host immune system interaction and role of co-receptors

The immune system can be activated by tumor antigens and, once primed, can elicit an antitumor response which in some cases can result in tumor destruction. Unfortunately, the successful development of antitumor immunity is often hampered by a plethora of factors that can directly determine the adequacy of the immune response. The singular event illustrated by a cytotoxic lymphocyte interacting with a tumor cell holds a background of a series of complex mechanisms, encompassed under the concepts of “immunosurveillance” and “immunoediting” ^8, 9^. Critical aspects in the tumor–immune system interface include the processing and presentation of released antigens by antigen-presenting cells (APCs), interaction with T lymphocytes, subsequent immune/T-cell activation, trafficking of antigen-specific effector cells, and, ultimately, the engagement of the target tumor cell by the activated effector T cell ^10, 11^. Nevertheless, although often successful in preventing tumor outgrowth, this “cancer-immunity cycle” can be disrupted by artifices involved in immune escape and development of tolerance, culminating with the evasion and proliferation of malignant cells ^9– 11^.

T-cell activation relies on the interaction of the T-cell receptor with antigens presented as peptides through the major histocompatibility complex (MHC) by the APC. Tumor antigens are classified as tumor-specific antigens (TSAs), derived from cancer-germline genes, point mutations or oncogenic viruses and unique to tumor cells, or tumor-associated antigens (TAAs), which include differentiation antigens (tyrosinase, gp100, Melan-A/MART-1, carcinoembryonic antigen, prostate-specific antigen, prostatic acidic phosphatase, etc.) and peptides associated with genes overexpressed in tumors (survivin, erbB-2 or CD340, RAGE-1, PRAME, and WT1) ^12, 13^. HLA downregulation has been shown to result in decreased antigenicity and therefore acts as a mechanism of immune evasion ^14^.

While the recognition of peptide–MHC by the TCR plays a central role in the process of T-cell-mediated immunity, additional cell-surface co-receptors are mandatory for the modulation of the immune response, either positively or negatively ^15, 16^. Two of these inhibitory co-receptors, called immune checkpoints, are involved in adaptive immune resistance and T-cell tolerance and have been exploited clinically with the development of checkpoint-blocking monoclonal antibodies. The two receptors include the cytotoxic T-lymphocyte-associated protein 4 (CTLA-4, also known as CD152) and the programmed cell death receptor 1 (PD-1 or CD279) and its ligand (PD-L1, also named CD274 or B7-H1) ^16^. Additional inhibitory receptors include B- and T-cell attenuator (BTLA or CD272), lymphocyte-activation protein 3 (LAG-3 or CD223), T-cell immunoglobulin and mucin protein-3 (TIM-3, also termed hepatitis A virus cellular receptor 2 – HAVCR2 – or CD366), and V-domain immunoglobulin-containing suppressor of T-cell activation (VISTA, B7H5, or programmed death 1 homolog – PD-1H) ^16– 18^. Also potential targets for therapeutic manipulation, co-stimulatory receptors associated with positive modulation of the immune synapse include CD27, CD28, CD137, inducible T-cell costimulator (ICOS or CD278), herpesvirus entry mediator (HVEM, also known as tumor necrosis factor receptor superfamily member 14 – TNFRSF14), and glucocorticoid-induced TNFR-related protein (GITR or tumor necrosis factor receptor superfamily member 18 – TNFRSF18). It is important to highlight, however, that the list of co-receptors and ligands encompasses both co-inhibitory and co-stimulatory molecules other than those aforementioned, some of which are not fully characterized.

The mobilization of these components of the adaptive immune system involved in antitumor immunity, including CD4+ helper T cells and CD8+ effector T cells, are largely influenced by a milieu of variables that involve intrinsic tumor characteristics, microenvironment factors, and genetic/epigenetic determinants ^19^.

## Tumor antigenic potential

Antigens are paramount in immune responses mediated by T cells; indeed, histologies that served as proofs of concept for the development of immunotherapy, including melanoma and renal cell carcinoma, have long been characterized as potentially “immunogenic” or “antigenic” ^19, 20^. Antigen-directed T-cell activation can result from the presentation of tumor self-peptides or peptides/neoantigens that emerge from aberrant gene products. As a consequence, the tumor genomic landscape or mutational load would represent a logical surrogate of the immunogenicity or “foreignness” of distinct malignancies through the generation of neoepitopes ^21, 22^.

Indeed, prolonged patient survival has been associated with an increased number of somatic missense mutations and mutational epitopes ^23^. More importantly, a correlation between the mutational burden and clinical benefit has been seen in the setting of immune-checkpoint blockade ^24– 26^. Snyder *et al*. was able to demonstrate an association between outcomes following anti-CTLA-4 therapy in melanoma and a high mutational load. Of note, although a high mutational load increased the probability of an “immunogenic” neoepitope signature, these variables were not completely overlapping. An even more intriguing finding was that candidate neoepitopes were homologous to distinct viral and bacterial antigens ^24^. Some similar findings were reported by Van Allen and colleagues based on an expanded cohort of 110 patients with metastatic melanoma; using transcriptome data, a correlation among the expression of cytolytic genes, neoantigen load, and clinical benefit to CTLA-4 was also demonstrated ^25^.

The mutational landscape was also found to be a determinant of clinical benefit from PD-1 blockade in patients with non-small-cell lung cancer (NSCLC); moreover, responses were more frequent in the setting of environmental exposure to tobacco, determined using a molecular signature of smoking that also correlated with a higher number of non-synonymous mutations ^26^. Serving as a strong proof of principle, blockade of PD-1 resulted in clinically meaningful activity in patients with mismatch repair (MMR) deficiency-associated tumors ^27^, characterized by a large number of somatic mutations and rich in expression of immune inhibitors (PD-1, PD-L1, LAG-3, and indoleamine 2,3 dioxygenase [IDO]) ^28^. Nevertheless, the correlation among mutational burden, the generation of neoantigens/neoepitopes, and the activation of antigen-specific T cells is not linear and neoepitopes may not be universally presented by the MHC ^29, 30^. Some studies have also suggested that mutational load may be prognostic but not necessarily predictive for responses to PD-1 therapy in melanoma ^31^. In addition, while clonal neoantigens may drive CD8+ T-cell responses and predict responses to PD-1 and CTLA-4 blockade, the clinical impact of subclonal mutations is largely debatable and arguably marginal, despite being associated with increased mutational load ^32^.

Albeit intuitive, the tumor antigenic potential is not driven solely by the total mutational load, as other antigens can also be immunogenic. Additional insults to the DNA other than the number of mutations can result in potentially neoantigenic epitopes, and oncogenic viruses could be determinant in the cancer–host immune system interaction and antigenicity. In Merkel cell carcinoma, the presence of Merkel cell polyomavirus (MCPyV) DNA and tumor-infiltrating lymphocytes and the expression of PD-L1 support the existence of intrinsic antitumor immunity ^33^. Indeed, PD-1 blockade resulted in meaningful clinical activity in Merkel cell carcinoma patients, particularly in those associated with MCPyV, despite a lower mutational burden in this subgroup ^4^.

While most studies have investigated the mutational profile of tumors as a surrogate for “tumor antigenicity” as a potential pre-treatment biomarker of responsiveness to checkpoint blockade, a recent study specifically examined the mutational profile of resistant lesions that arose in patients with melanoma who previously benefitted from PD-1 therapy. Although the number of patients examined in this series was small (n=4), some secondary resistant lesions had mutations in the interferon (IFN) (JAK mutations) and antigen-presentation (beta-2-microglobulin) pathways, suggesting possible mechanisms of immune escape from PD-1 ^34^. Additional study in larger patient cohorts would be of value.

## Tumor microenvironment factors and pre-existing host immune conditions

Despite the central role of intrinsic antigenicity, tumor immunogenicity is directly influenced by a plethora of immunomodulatory factors co-existing in the tumor microenvironment that derive from both tumor cells and host cells. Also intuitive, the concept that “inflamed” or “hot” tumors may derive greater benefit from immunotherapy is supported by mounting evidence.

The characterization of the T-cell infiltrate has been associated with both innate antitumor immunity and benefit from immune-checkpoint blockade. The density of antigen-specific effector T cells within the tumor microenvironment and invasive margin is a predictor of survival in patients with colorectal cancer, and the concept that pre-treatment adaptive immune responses and immune infiltrates directly influence the natural course of different malignancies is consistent across different studies ^35, 36^. Pre-existing CD8+ T cells located at the invasive tumor margin are aligned with expression of PD-1 and PD-L1 ^37^. Additionally, increased CD8+ T-cell infiltrates within the tumor microenvironment directly correlated with benefit from PD-1 blockade ^37, 38^. An association between absolute lymphocyte count in the peripheral blood of patients who received anti-CTLA-4 therapy and clinical benefit has also been shown as an increase in lymphocyte count during treatment or at baseline ^39, 40^. Also, anti-CTLA-4 treatment was demonstrated to result in newly detected CD8+ T-cell responses measured in post-treatment samples, suggesting that CTLA-4 blockade has a direct role in increasing T-cell priming ^41^.

Increased levels of IFN-γ and expression of ICOS on peripheral lymphocytes and tumor-infiltrating lymphocytes has been demonstrated in the setting of CTLA-4 blockade ^42^, providing the rationale for additional combined approaches. CD4+ T cells with increased ICOS expression also correlated with an increase in effector/regulatory T-cell ratio ^43^.

Factors involved in the modulation of the tumor and immune microenvironment are also crucial in understanding the tumor–host immune system interaction. In metastatic melanoma samples, cell lines, and xenografts, T-cell and macrophage recruitment occurred more frequently in association with the expression of a subset of chemokines (CCL2, CCL3, CCL4, CCL5, CXCL9, and CXCL10) associated with an “inflamed” phenotype ^44, 45^. Indeed, CXCL9 and CXCL10, ligands of CXCR3, were incorporated in a gene signature associated with responses to anti-PD-1 treatment and indicative of an inflamed microenvironment ^46^. Similarly, the presence of tumor-reactive cells correlated with endogenous accumulation of type I IFNs (IFN-α, IFN-β, IFN-ε, IFN-κ, and IFN-ω) ^47^. In a topic of significant clinical relevance, the regulation of genes associated with IFN signaling was achieved with the use of azacitidine, a DNA methyltransferase inhibitor, through the epigenetic regulation of gene promoters normally silenced ^48^.

Nevertheless, T-cell infiltration is also accompanied by the induction of tolerance mechanisms largely involved in the abrogation of an effective antitumor immune response. These so-called inhibitory pathways involve the expression of IDO and PD-L1, induced by IFN-γ, and recruitment of FoxP3+CD4+ (regulatory T) cells through CCL22 in the setting of CD8+ T-cell activation ^49^.

In melanoma lesions and other malignancies, the expression of PD-L1 has been associated with the presence of tumor-infiltrating lymphocytes, IFN-γ expression, and improved survival in some studies ^33, 50– 52^. While straightforward, using the expression of PD-L1 as a biomarker poses a series of caveats and uncertainties. PD-L1 is expressed in macrophages and, in the setting of immune activation, in B, T, myeloid, and dendritic cells (DCs) as well as in non-hematopoietic and endothelial cells ^53^. Indeed, the early clinical development of anti-PD-1 agents already suggested that tumors rich in PD-L1 expression were more likely to respond to therapy ^54^, although this correlation is imperfect. It is important to emphasize that PD-L1 expression occurs along a spectrum of positivity and is dynamic and heterogeneous between and within tumors. The expression of PD-L1 can occur constitutively, or it can be induced upon T-cell activation ^49, 50, 55^. In addition to analytical technical issues detecting PD-L1, pre-testing factors (distribution, cell population by which PD-L1 is expressed, etc.) and intrapatient, intertumor heterogeneity pose significant limitations to the interpretation of PD-L1 expression ^56^.

Similarly, the expansion of regulatory T cells and myeloid-derived suppressor cells (MDSCs) also represents a mechanism of immune escape, suggesting that additional immunosuppressive factors may need to be targeted to increase antitumor immunity. In murine models, inhibition of MDSC trafficking by CXCR2 deficiency or CXCR2 signaling blockade increased the efficacy of anti-PD-1 therapy ^57^. It has been demonstrated in pre-clinical models that inhibition of regulatory T cells may be necessary for anti-CTLA-4-induced antitumor activity ^58^. Moreover, the efficacy of anti-CTLA-4 therapy has been associated with regulatory T-cell depletion in the presence of Fcγ receptor-expressing macrophages, suggesting that the mechanisms involved in immune activation may be more diverse than anticipated ^59^.

In addition to the aforementioned factors related to pre-existing immune conditions and regulation within the tumor microenvironment, antitumor immunity can also be affected by a very particular variable: the host microbiota. Across different studies, intestinal commensal bacteria have been shown to influence T-cell differentiation, APC activation, and antitumor immunity modulation ^60, 61^. In a demonstration of this principle, fecal material transfer between two murine populations resulted in infiltration of tumor-specific CD8+ T cells and delayed tumor growth, an effect attributed to the colonization by Bifidobacterium species. Of note, in addition to innate antitumor immunity, oral administration of Bifidobacterium also potentiated the antitumor effect of PD-L1 blockade ^62^.

## Genetic, epigenetic, and signaling modulators of the immune response

If it is now well established that immune responses can be influenced by genomic correlates, including the burden of non-synonymous mutations, emerging evidence suggests that specific genetic variables are also involved in direct modulation of antitumor immunity.

Distinct somatic mutations have been shown to be related to intratumoral immunity. As an example, restoration of p53 signaling has been associated with the activation of tumor-directed innate immune cells, natural killer cell recruitment, and chemokine production ^63, 64^. Another study showed a low mutational burden was associated with PD-L1 negativity and worse survival ^65^. In melanoma cell lines, disruptive mutations of JAK1 or JAK2 (downstream elements of IFN signaling) have been shown to abrogate PD-L1 expression upon exposure to IFN-γ, suggesting a mechanism for innate resistance to PD-1 blockade ^66^. In NSCLC samples, expression of PD-L1 and PD-L1 gene amplification correlated with simultaneous amplification of JAK2, whereas JAK2 inhibition resulted in reduced expression of PD-L1 protein ^67^. Conversely, PI3K-AKT pathway activation resulting from PTEN loss has been shown to correlate with immunoresistance mediated by PD-L1 and PD-L2 expression in preclinical models as well as in a clinical series ^55, 68^. An immune translation of somatic events has also been reported for aberrations involving STAT3/ALK signaling and EGFR mutations ^69, 70^. In melanoma metastases, mutations involved in activation of the WNT/β-catenin pathway were associated with a non-T-cell inflamed phenotype and T-cell exclusion from the tumor microenvironment ^71^.

Besides T-cell activation, DC mobilization can be modulated by distinct genetic pathways involved in innate immune sensing of immunogenic tumors. As an example, knockout mice deficient for the transcription factor Batf3, involved in DC recruitment and activation through type I IFNs, show impaired CD8+ T-cell activation ^72^. As a corollary, tumor-infiltrating DCs can be artificially manipulated in order to induce antitumor immunity, as demonstrated in preclinical models in which intratumoral delivery of mRNA involved in the activation of cross-presenting DCs resulted in T-cell responses ^73^. Similarly, defective spontaneous T-cell priming has been demonstrated in models lacking the cytosolic receptor stimulator of IFN genes complex (STING), which is involved in type I IFN and proinflammatory cytokine responses. In line with these observations, vaccines with STING ligands were able to induce DCs, PD-L1 upregulation, and antigen-specific T-cell activation in preclinical models ^74^.

## Conclusions

It is well known that the characterization of basic mechanisms underlying antitumor immunity has paved the way for the development of therapeutic strategies to manipulate antitumor immunity for favorable patient benefit. The interplay among different factors driving the tumor–host immune response is not fully characterized, and the complexity of these factors has been summarized by Blank and colleagues as the “cancer immunogram” ^75^. The understanding of these multiple regulatory pathways involved in antitumor immunity is crucial not only for patient selection and therapeutic decisions but also for improving outcomes through combined approaches. In addition, despite the significant clinical results and survival improvements seen in patients with some cancers, primary and acquired/secondary resistance to immunotherapy remain challenges. Future research will be critical in addressing the large body of questions which remains to be answered.
